# Long COVID services: Implementing discrete event simulation, a quality improvement proposition

**DOI:** 10.1016/j.clinme.2024.100280

**Published:** 2024-12-12

**Authors:** Eman Albastaki

**Affiliations:** The Dudley Group NHS Foundation Trust, Dudley, West Midlands, United Kingdom

Dear Editor,

I thoroughly enjoyed reading the article by Darbyshire *et al*, which aimed to enhance the multidisciplinary approach needed to manage long COVID cases. The study was conducted in 10 sites already involved in the wider LOCOMOTION study across England. A holistic care was designed to address all aspects of long-term COVID complaints. To overcome the little literature published on this topic, frequent quality improvement trajectories were structured across the study from 2021 to 2023, created through recurrent meetings to discuss guidelines, cases and clinic audits.^1^

I first want to thank the authors for their valuable work, which will contribute immensely to the knowledge needed to provide holistic care for patients with long COVID.

One of the difficulties that the authors mentioned was the expanding waiting list of patients due to a backlog of referrals. The authors attributed the issue to multiple factors, which they tried and succeeded in addressing, thus decreasing the waiting duration for assessment. However, the authors explained that overcoming this dilemma is still challenging, with new cases not meeting the standard period.

One of the comprehensive methods to deal with such a problem would be a discrete event simulation tool. Such simulation would help institutions define their bottlenecks using special software to elude reality without causing any serious implications.^2^ A study conducted in the UK used an endoscopy dataset of patients on the waiting list at Cambridge University Hospitals NHS Foundation Trust following the COVID pandemic.^3^ They used a discrete event simulation model to estimate the average waiting list delay due to the prioritisation system.^3^ Therefore, the article suggested testing different scenarios according to patients’ specific characteristics to allow for a better prediction of the overall outcomes.^3^

Another study has proven the efficacy of implementing a discrete event simulation model when dealing with long waiting hours in the emergency department (ED).^4^ The model effectively identified subtle contributing factors and predicted the results of equipment and resource modifications. The model succeeded in improving the length of stay below 3 h to 80%.^4^

In conclusion, implementing discrete event simulation models might be a potential solution when dealing with long waiting lists to improve the quality of service.

## Funding

This letter did not receive any specific grant from funding agencies in the public, commercial or not-for-profit sectors.

## CRediT authorship contribution statement

**Eman Albastaki:** Conceptualization, Writing – original draft, Writing – review & editing.

## Declaration of competing interest

The author declares that they have no known competing financial interests or personal relationships that could have appeared to influence the work reported in this paper.
